# Resveratrol Trimers from Seed Cake of *Paeonia rockii*

**DOI:** 10.3390/molecules191219549

**Published:** 2014-11-26

**Authors:** Pu Liu, Yiran Wang, Jiayu Gao, Zongyuan Lu, Weiping Yin, Ruixue Deng

**Affiliations:** Chemical Engineering & Pharmaceutical College, Henan University of Science and Technology, Research Center on Wild Natural Resources of FUNIU Mountain, Luoyang 471023, China; E-Mails: liuputju@163.com (P.L.); gjiayu@126.com (Y.W.); cruise1204@163.com (J.G.); zuyuang@126.com (Z.L.)

**Keywords:** seed cake, *Paeonia rockii*, resveratrol trimers, antibacterial activity

## Abstract

In the course of screening natural products for antibacterial activities, a total acetone extract of the seed cake of *Paeonia rockii* showed significant effects against bacterial strains. Bioactivity-guided fractionation of the EtOAc-soluble fraction of the total acetone extract resulted in the isolation and identification of five resveratrol trimers, including rockiiol C (**1**), gnetin H (**2**), suffruticosol A (**3**), suffruticosol B (**4**) and suffruticosol C (**5**). The relative configuration of these compounds was elucidated mainly by comprehensive 1D and 2D-NMR experiments. Compound **1** was a new compound. All isolated compounds exhibited strong antibacterial activities against Gram-positive bacteria.

## 1. Introduction

*Paeonia rockii* subsp. *rockii* cv “spp.” (Paeoniaceae) is a shrub widely distributed in China. The seeds oil (peony seed oil) from seeds of *Paeonia ostii* or *Paeonia rockii* has been authorized as a new resource food by the Ministry of Health of the People’s Republic of China (Bulletin of the Ministry of Health of the People’s Republic of China announced, 2011, No. 9). The seed cake is the major by-product in the preparation procedure of crushing cooking oil from the seeds of *Paeonia rockii*. Though no reports were found regarding the chemical constituents of the seed cake of *P. rockii*, studies on the seeds of other *Paeonia* genera have been performed elsewhere [1,2,3,4,5,6,7]. 

**Figure 1:**
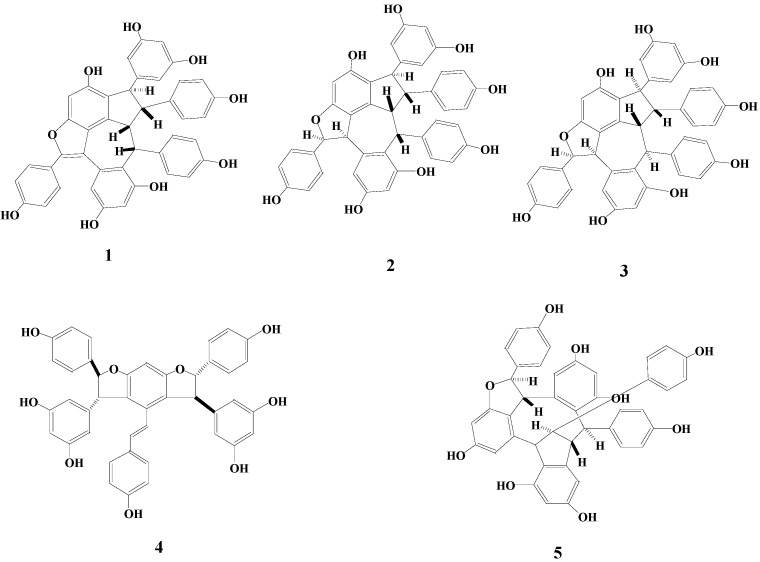
Structures of Compounds **1**–**5**.

In the course of screening natural products for antibacterial activities, it was found that the acetone extract from the seed cake of *P. rockii* had significant inhibitory effects on bacterial strains. Further bioactivity-guided fractionation led to the isolation and identification of one new resveratrol trimer, rockiiol C (**1**), together with the four known ones. Their structures are depicted in Figure 1. Herein, we mainly describe the isolation and structural elucidation of the new resveratrol trimer on the basis of various 2D-NMR techniques, including HSQC, HMBC, ^1^H-^1^H COSY and NOESY experiments. 

## 2. Results and Discussion

### 2.1. Purification and Characterization

The EtOAc-soluble fraction of the acetone extract from the seed cake of *P. rockii* was separated by silica-gel, gel permeation chromatography and pre-HPLC (ODS-A) to give one new resveratrol trimer, rockiiol C (**1**), and four known compounds (**2**–**5**) (Figure 1). 

### 2.2. Structural Elucidation of Compound **1**

Compound **1**, was obtained as a brownish-white amorphous powder and showed a positive reaction with Gibbs reagent. The HRESI-MS data (pseudomolecular ion [M+H]^+^ at *m*/*z* 679.1965 (calcd. 679.1970)) together with the ^13^C-NMR data (42 carbons) indicated the molecular formula C_42_H_3__0_O_9_, which suggested that **1** was a resveratrol trimer. The IR spectrum of Compound **1** revealed the presence of hydroxy (3336 cm^−1^) and aromatic rings (1632, 1515 and 1450 cm^−1^). The UV spectrum displayed an absorption maximum at 289 nm, which was consistent with one or more non-conjugated phenyl rings.

The ^1^H- and ^13^C-NMR spectrum data (Table 1) exhibited six sets of AX-type hydrogen (δ 7.41 (d, *J* = 8.8 Hz), δ 6.75 (d, *J* = 8.8 Hz), δ 6.68 (d, *J* = 8.5 Hz), δ 6.26 (d, *J* = 8.5 Hz), δ 6.17 (d, *J* = 8.7 Hz), δ 6.02 (d, *J* = 8.7 Hz)) two sets of *meta*-coupled aromatic hydrogen (δ 6.54 (d, *J* = 2.5 Hz), δ 6.20 (d, *J* = 2.5 Hz), δ 6.08 (d, *J* = 2.1 Hz), δ 6.04 (d, *J* = 2.1 Hz)) and singlet aromatic hydrogen (δ 6.82 (s)). Compound **1** showed four aliphatic signals at δ 5.25 (d, *J* = 3.3 Hz), δ 4.35 (dd, *J* = 3.3, 9.1 Hz), δ 3.78 (dd, *J* = 1.5, 9.1 Hz) and δ 4.62 (d, *J* = 1.5 Hz), whereas Compounds **2** and **3**, exhibited six aliphatic hydrogens. Complete assignment of all protons and carbons was confirmed by ^13^C-^1^H COSY. All one- and two-dimensional NMR spectroscopic analyses data indicated that Compound **1** is a dehydro derivative of Compounds **2** and **3**. In comparison, for Compounds **2** and **3**, a similar HMBC correlation pattern was revealed in Compound **1**, although some differences arose from the unsaturated benzofuran structure. The spectrum data of Compound **1** were similar to that of melapinol B [8], which suggested that **1** was also an oxidative trimer of resveratrol, and the planar structure was the same as that of melapinol B. The planar structure of **1** was confirmed by the one- and two-dimensional NMR spectroscopic analyses data. The spectrum data of the partial structure (C1-C2 and B1-B2) of Compound **1** were similar to those of melapinol B [8], which indicated that they have the same partial structure. 

**Table 1:** ^1^H- and ^13^C-NMR data of **1**, together with HMBC (H→C) correlations. At 400/100 MHz, respectively, in CD_3_OD; δ in ppm, *J* in Hz.

No.	^1^H δ *J* (Hz)	^13^C	HMBC	No.	^1^H δ *J* (Hz)	^13^C	HMBC
**1**		135.1		**8'**	4.35 dd, 3.3, 9.1	51.8	9',10',1'
**2**	6.68 d, 8.5	130.8	7,1,4	**9'**		141.5	
**3**	6.26 d, 8.5	115.0	1,4	**10'**		120.6	
**4**		156.2		**11'**		154.6	
**5**	6.26 d, 8.5	115.0		**12'**	6.82 s	96.4	10',11',13',14'
**6**	6.68 d, 8.5	130.8		**13'**		153.0	
**7**	3.78 dd,1.5,9.1	61.7	2,8,9,7'	**14'**		127.9	
**8**	4.62 d, 1.5	58.1	7,9,10,8',14'	**1''**		124.7	
**9**		150.0		**2''**	7.41 d, 8.8	130.9	4'',6'',7''
**10**	6.08 d, 2.1	106.6	8,12,14	**3''**	6.75 d, 8.8	116.3	1'',4'',5''
**11**		159.3		**4''**		159.1	
**12**	6.04 t, 2.1	101.3	10,11	**5''**	6.75 d, 8.8	116.3	4''.2'',7''
**13**		159.3		**6''**	7.41 d, 8.8	130.9	1'',4'',3''
**14**	6.08 d, 2.1	106.6		**7''**		152.4	2'',9''
**1'**		136.4		**8''**		116.4	1'',10',7''
**2'**	6.17 d, 8.7	131.2	7',6',4'	**9''**		141.2	
**3'**	6.02 d, 8.7	114.6	1',4'	**10''**		126.3	
**4'**		154.6		**11''**		155.7	
**5'**	6.02 d, 8.7	114.6	1',4'	**12''**	6.20 d, 2.5	102.5	10'',11'',14''
**6'**	6.17 d, 8.7	131.2	7',2',4'	**13''**		155.7	
**7'**	5.25 d, 3.3	42.1	1',2',8',9',9'',10''	**14''**	6.54 d, 2.5	110.0	8'',10'',12'',13''

**Figure 2:**
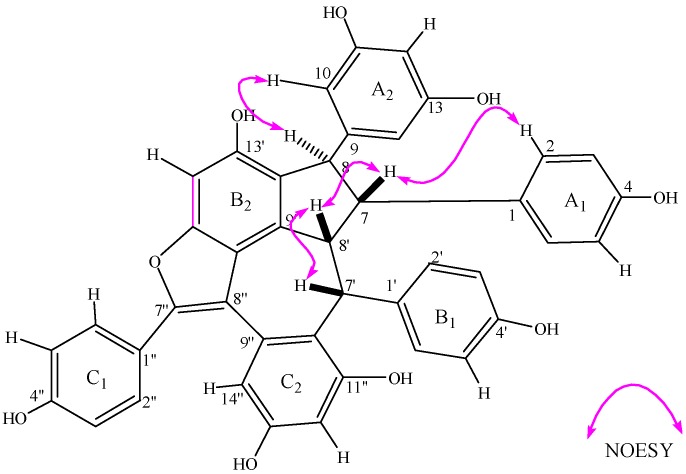
The key NOESY correlations of Compound **1**.

The NOESY (Figure 2) cross peaks between H-7'/H-8' indicated that the rings, H-7' and H-8', were in the β-configuration as in melapinol B [8]. The significant NOEs between H-8'/H-7 indicated that the relative configuration of the methine proton at C-7 was β-configuration. Compound **1** showed NOE interactions between H-8 and H-10, between H-7 and H-2(6) and between H-7 and H-8', which is proof of the trans-configuration between H-7 and H-8. Therefore, the methine proton at C-8 was in the α-configuration. The assignment of the relative configuration of **1** was *7R*, *8R*, *7'R*, *8'R*, according to the NOE correlations of Compound **1** and the configuration of melapinol B. Therefore, the structure of **1** was designated as shown in Figure 1.

The other known resveratrol oligomers were isolated, and their structures were identified as suffruticosol A (**2**) [2], suffruticosol B (**3**) [2], gnetin H (**4**) [9] and suffruticosol C (**5**) [2].

### 2.3. Antibacterial Activities

The *in vitro* anti-bacterial effects of the isolated compounds (**1**–**5**) were tested. It was found that all of these compounds exhibited strong antibacterial activities against Gram-positive bacteria. The MIC values against bacterial strains of Compounds **1**–**5** are listed in Table 2. 

**Table 2:** The antibacterial activities of the compounds (MIC, μg/mL).

Compound	*Staphylococcus aureus*	*Pyogenic streptococcus*	*Streptococcus viridans*	*Staphylococcus epidermidis*	*Escherichia coli*	*Pseudomonas aeruginosa*
Penicillin G	10	10	10	10	20	10
**1**	20	20	25	20	200	200
**2**	20	20	25	20	200	100
**3**	20	20	25	25	100	200
**4**	25	25	25	20	200	200
**5**	25	30	20	25	100	100

## 3. Experimental Section

### 3.1. General Experimental Procedures

Optical rotation was measured with a MC 241 digital polarimeter (Perkin-Elmer, Waltham, MA, USA). IR spectra were recorded on a Perkin-Elmer-577 spectrometer infrared Fourier transform spectrometer (Perkin-Elmer, Waltham, MA, USA). NMR spectra were performed on a Bruker AVANCE 400 instrument with tetramethylsilane as an internal standard (Rheinstetten, Germany), at 400 (^1^H) and 100 MHz (^13^C). HR-ESI-MS was obtained on a Waters LCT-Premier instrument (Milford, MA, USA). HPLC was performed using a Waters 600 with Waters TP pump, UV-2487 detector (Milford, MA, USA) and an YMC-Pack ODS-A column (SH-343-5, Tokyo, Japan). Column chromatography (CC) was performed on silica gel (Qingdao Marine Chemical Co., Ltd, Qingdao, China), Toyopearl HW-40 (TOSOH, Tokyo, Japan). Thin-layer chromatography (TLC) was performed on silica gel GF_254_ plates (Qingdao Marine Chemical Co., Ltd, Qingdao, China), visualization under UV light and by spraying with Ce_2_SO_4_ or phosphomolybdic acid hydrate, followed by heating.

### 3.2. Plant Material

The seeds of *Paeonia rockii* were collected at Luoyang Tuqiao flower and seeding CO. LTD, Luoyang city, Henan province, and were verified by Hou Xiao-gai (College of Agricultural, Henan University of Science and Technology) in September, 2012. A voucher specimen has been deposited in the Specimens Hall of Natural resources of Funiu Mountains, Henan University of Science and Technology. The seed cake was obtained in the preparation procedure of crushing cooking oil from the seeds.

### 3.3. Extraction and Isolation

Air-dried seed cakes of *P. rockii* (7.5 kg) were extracted by acetone at room temperature, and 500 g of dry crude residues remained after solvent evaporation *in vacuo*. The residues were dissolved in methanol, and the methanol dissolved fraction (300 g) was then added into the mixtures (1000 g) of silica and diatomite (1:1, W/W). The whole sample was dried at room temperature and then was fractionated successively with EtOAc and methanol to produce 110-g and 150-g dried fractions, respectively. The EtOAc-soluble fraction of the acetone extract was further repeatedly chromatographed on a silica gel column using a gradient solvent system (petroleum ether (PE)–acetone (8:1, 4:1, 2:1, 1:1)) to obtain 10 fractions. 

Fraction 5 (14.4 g) was chromatographed on CC to get five subfractions. Subfraction 5.4 (1400 mg) was separated by semi-preparative HPLC (ODS, MeOH–H_2_O, 50:50) to obtain Compound **2** (23.1 mg). 

Fraction 6 (8.6 g) was separated on semi-preparative HPLC (ODS, MeOH–H_2_O, 45:55) to afford six subfractions. Compound **3** (12.3 mg) and Compound **4** were obtained by HPLC (ODS-A; MeCN–H_2_O 2:8, 3.0 mL/min) from Subfraction 6.1 (2,780 mg), and Compounds **1** (9.8 mg) and **5** (300.3 mg) were isolated from Subfraction 6.5 (780 mg).

Rockiiol C (**1**): Brownish-white amorphous powder. ${\left[\alpha \right]}_{D}^{25}$ = −78.2 (c 0.62, MeOH). UV (MeOH) λ_max_ (logε): 289 (3.57), 228 (3.98). IRν_max_ (KBr) cm^−1^: 3,336, 1,632, 1,515 and 1,450. The ^1^H-NMR (CD_3_OD, 400 MHz) and ^13^C-NMR (CD_3_OD, 100 MHz) see Table 1. HRESI-MS: *m*/*z* 679.1965 [M+H]^+^ (calcd. for 679.1970). 

Suffruticosol A (**2**): Brownish-white amorphous powder, mp 293–296 °C, ESI-MS *m/z*: 679 [M−H]^−^. ^1^H-NMR (CD_3_OD, 400 MHz), δ: 7.10 (2H, d, *J* = 8.6 Hz, H-2', 6'), 6.68 (2H, d, *J* = 8.6 Hz, H-3', 5'), 6.47 (2H, d, *J* = 8.6 Hz, H-2'', 6''), 6.11 (2H, d, *J* = 8.6 Hz, H-3'', 5''), 6.95 (2H, d, *J* = 8.2 Hz, H-2, 6), 6.36 (2H, d, *J* = 8.2 Hz, H-3, 5), 6.25 (1H, d, *J* = 1.68 Hz, H-14''), 5.92 (1H, d, *J* = 1.68 Hz, H-12''), 6.19 (1H, d, *J* = 0.56 Hz, H-12'), 6.05 (1H, t, *J* = 2.16 Hz, H-12), 5.97 (2H, t, *J* = 2.16 Hz, H-10, 14), 3.67 (1H, d, *J* = 7.72 Hz, H-7), 4.74 (1H, brs, H-8), 5.42 (1H, d, *J* = 3.24 Hz, H-7'), 3.93 (1H, m, H-8'), 5.68 (1H, d, *J* = 15.7 Hz, H-7''), 4.34 (1H, d, *J* = 15.7 Hz, H-8''). ^13^C-NMR(CD_3_OD, 100 MHz) δ: 135.6 (C-1), 130.9 (C-2), 115.5 (C-3), 156.5 (C-4), 115.5 (C-5), 130.9 (C-6), 60.7 (C-7), 54.6 (C-8), 148.4 (C-9), 106.8 (C-10), 159.3 (C-11), 101.4 (C-12), 159.3 (C-13), 106.8 (C-14), 133.9 (C-1'), 130.5 (C-2'), 116.3 (C-3'), 159.0 (C-4'), 116.3 (C-5'), 130.5 (C-6'), 39.7 (C-7'), 49.1 (C-8'), 144.7 (C-9'), 117.3 (C-10'), 160.2 (C-11'), 96.2 (C-12'), 155.0 (C-13'), 123.0 (C-14'), 130.9 (C-1''), 130.8 (C-2''), 114.2 (C-3''), 154.5 (C-4''), 114.2 (C-5''), 130.8 (C-6''), 91.5 (C-7''), 48.6 (C-8''), 141.8 (C-9''), 126.9 (C-10''), 155.2 (C-11''), 105.9 (C-12''), 156.7 (C-13''), 101.9 (C-14'').

Suffruticosol B (**3**): Brownish-white amorphous powder, mp 297–301 °C, ESI-MS *m*/*z*: 679 [M−H]^−^. ^1^H-NMR (CD_3_OD, 400 MHz), δ: 6.95 (2H, d,* J* = 8.6 Hz, H-2', 6'), 6.49 (2H, d, *J* = 8.6 Hz, H-3', 5'), 7.57 (2H, d, *J* = 8.6 Hz, H-2'', 6''), 6.93 (2H, d, *J* = 8.6 Hz, H-3'', 5''), 6.27 (2H, d, *J* = 8.4 Hz, H-2, 6), 6.29 (2H, d, *J* = 8.2 Hz, H-3, 5), 5.94 (1H, d, *J* = 2.16 Hz, H-14''), 6.17 (1H, d, *J* = 2.16 Hz, H-12''), 6.19 (1H, s, H-12'), 6.14 (1H, t, *J* = 2.16 Hz, H-12), 6.22 (2H, t, *J* = 2.16 Hz, H-10, 14), 3.80 (1H, d, *J* = 6.0 Hz, H-7), 4.08 (1H, m, H-8), 4.22 (1H, d, *J* = 11.72 Hz, H-7'), 4.10 (1H, brd, *J* = 11.76, H-8'), 5.86 (1H, d, *J* = 11.84 Hz, H-7''), 5.08 (1H, d, *J* = 11.84 Hz, H-8'').^13^C-NMR(CD_3_OD, 100 MHz) δ: 135.5 (C-1), 129.5 (C-2), 115.2 (C-3), 156.1 (C-4), 115.2 (C-5), 129.5 (C-6), 63.1 (C-7), 56.9 (C-8), 147.5 (C-9), 107.4 (C-10), 159.4 (C-11), 101.5 (C-12), 159.4 (C-13), 107.4 (C-14), 133.8 (C-1'), 133.1 (C-2'), 114.7 (C-3'), 156.1 (C-4'), 114.7 (C-5'), 133.1 (C-6'), 46.5 (C-7'), 47.8 (C-8'), 144.7 (C-9'), 117.3 (C-10'), 160.2 (C-11'), 96.2 (C-12'), 155.0 (C-13'), 123.6 (C-14'), 130.9 (C-1''), 130.6 (C-2''), 116.5 (C-3''), 159.2 (C-4''), 116.5 (C-5''), 130.6 (C-6''), 91.2 (C-7''), 49.0 (C-8''), 142.4 (C-9''), 122.9 (C-10''), 157.2 (C-11''), 105.0 (C-12''), 1,558.4 (C-13''), 103.7 (C-14'').

Gnetin H (**4**): White powders, mp 185–187 °C, ESI-MS *m*/*z*: 679 [M−H]^−^. ^1^H-NMR (CD_3_OD, 400 MHz), δ: 7.19 (4H, dd, *J* = 2.0, 8.6 Hz, H-2, 6, 2'', 6''), 6.79 (4H, dd, *J* = 2.0, 8.6 Hz, H-3, 5, 3'', 5''), 6.69 (2H, dd, *J* = 2.0, 8.6 Hz, H-2', 6'), 6.51 (2H, dd, *J* = 2.0, 8.6 Hz, H-3', 5'), 6.43 (1H, s, H-12'), 6.38 (2H, brs, H-7', 8'), 6.14 (6H, s, H-10, 12, 14, 10'', 12'', 14''), 5.41 (2H, d, *J* = 5.64 Hz, H-7, 7''), 4.40 (2H, d, *J* = 5.64 Hz, H-8, 8'').^13^C-NMR(CD_3_OD, 100 MHz) δ: 134.2 (C-1), 128.1 (C-2), 116.4 (C-3), 158.6 (C-4), 116.4 (C-5), 128.1 (C-6), 94.5 (C-7), 59.0 (C-8), 147.6 (C-9), 107.3 (C-10), 160.1 (C-11), 102.1 (C-12), 160.1 (C-13), 107.3 (C-14), 130.6 (C-1'), 128.7 (C-2'), 116.2 (C-3'), 158.3 (C-4'), 116.2 (C-5'), 128.7 (C-6'), 134.6 (C-7'), 122.5 (C-8'), 128.1 (C-9'), 120.3 (C-10'), 163.0 (C-11'), 91.5 (C-12'), 163.0 (C-13'), 120.3 (C-14'), 133.7 (C-1''), 128.1 (C-2''), 116.4 (C-3''), 158.6 (C-4''), 116.4 (C-5''), 128.1 (C-6''), 94.9 (C-7''), 59.0 (C-8''), 147.6 (C-9''), 107.3 (C-10''), 160.1 (C-11''), 102.1 (C-12''), 160.1 (C-13''), 107.3 (C-14''). 

Suffruticosol C (**5**). Brownish-white amorphous powder, mp 197–199 °C, ESI-MS *m*/*z*: 679 [M−H]^−^. ^1^H-NMR (CD_3_OD, 400 MHz), δ: 7.20 (2H, d, *J* = 8.4 Hz, H- 2', 6'), 6.78 (2H, d, *J* = 8.4 Hz, H-3', 5'), 7.08 (2H, d, *J* = 8.4 Hz, H-2'', 6''), 6.78 (2H, d, *J* = 8.4 Hz, H-3'', 5''), 6.92 (2H, d, *J* = 8.4 Hz, H-2, 6), 6.51 (2H, d, *J* = 8.4 Hz, H-3, 5), 6.38 (1H, d, *J* = 2.24 Hz, H-10''), 6.23 (1H, d, *J* = 2.24 Hz, H-12''), 5.98 (1H, s, H-12'), 6.00 (1H, t, *J* = 2.2 Hz, H-12), 5.86 (2H, t, *J* = 2.2 Hz, H-10, 14), 4.28 (1H, d, *J* = 10.2 Hz, H-8'), 2.97 (1H, m, H-7'), 4.14 (1H, brd, *J* = 11.72, H-8), 6.00 (1H, d, *J* = 1.34 Hz, H-7''), 4.23 (1H, d, *J* = 1.34 Hz, H-8''), 5.07 (1H, brs, H-7). ^13^C-NMR(CD_3_OD, 100 MHz) δ: 138.0 (C-1), 129.8 (C-2), 115.5 (C-3), 155.8 (C-4), 115.5 (C-5), 129.8 (C-6), 36.8 (C-7), 52.3 (C-8), 144.0 (C-9), 123.1 (C-10), 158.8 (C-11), 101.3 (C-12), 158.8 (C-13), 107.8 (C-14), 133.0 (C-1'), 130.9 (C-2'), 115.9 (C-3'), 156.9 (C-4'), 115.9 (C-5'), 133.0 (C-6'), 67.0 (C-7'), 57.7 (C-8'), 147.2 (C-9'), 107.8 (C-10'), 159.6 (C-11'), 96.2 (C-12'), 155.0 (C-13'), 1119.5 (C-14'), 134.8 (C-1''), 128.0 (C-2''), 116.2 (C-3''), 158.2 (C-4''), 116.2 (C-5''), 128.0 (C-6''), 86.3 (C-7''), 51.0 (C-8''), 147.6 (C-9''), 103.5 (C-10''), 157.1 (C-11''), 101.3 (C-12''), 158.9 (C-13''), 118.6 (C-14'').

### 3.4. Bioassay

The MIC (minimum inhibitory concentration) of Compounds **1**–**5** against the bacterial strains *Staphylococcus aureus*, pyogenic streptococcus, *Streptococcus viridans*, *Staphylococcus epidermidis*, *Escherichia coli* and *Pseudomonas aeruginosa*, were determined using the standard broth microdilution method [10,11]. Penicillin G was used as the positive control. The test compounds (50.0 mg) were diluted in Me_2_SO (500 μL) and mixed with bacterial strains cultured in nutrient broths (9.5 mL). The initial concentration of test compounds was 5 mg/mL, and concentrations of 10, 20, 30, 40, 50, 60, 70, 80, 100, 200, 300, 400 and 500 μg/mL were obtained by serial dilutions. Antibacterial tests were performed by transferring each test compound into different concentration (1 mL) into a new test tube containing only bacterial culture (1 mL). Observations were made after 24 h to determine the possible bacterial growth in the respective culture broths. The optical density of treated cells reflects their viability and provides sufficient information pertaining to the mode of action of the tested metabolites.

## 4. Conclusions

In summary, one new compound, **1**, and four known compounds (**2**–**5**) were characterized from the seed cake of *Paeonia rockii*. These compounds could be classified into resveratrol trimers. Additionally, the basic unit of these compounds was resveratrol. The results revealed that these compounds displayed varying degrees of antibacterial activity. The isolated compounds were all oligostilbenes. Oligostilbenes are a class of plant polyphenols and have attracted intense interest for their intricate structures and diverse biological activities. These compounds and their derivatives are of significant interest for drug research and development, because of their potential in therapeutic or preventive applications, such as anti-bacterial agents, antioxidant and antitumor drugs, and so on. 

## Supplementary Materials

Supplementary materials can be accessed at: http://www.mdpi.com/1420-3049/19/12/19549/s1.
