# Supplementary material for: Resveratrol Trimers from Seed Cake of Paeonia rockii
Source: Molecules. 2014 Nov 26;19(12):19549–56. doi: 10.3390/molecules191219549 (PMC6271524; doi:10.3390/molecules191219549)

Supplementary Materials

Figure S1. ^{1}H-NMR of **1**.

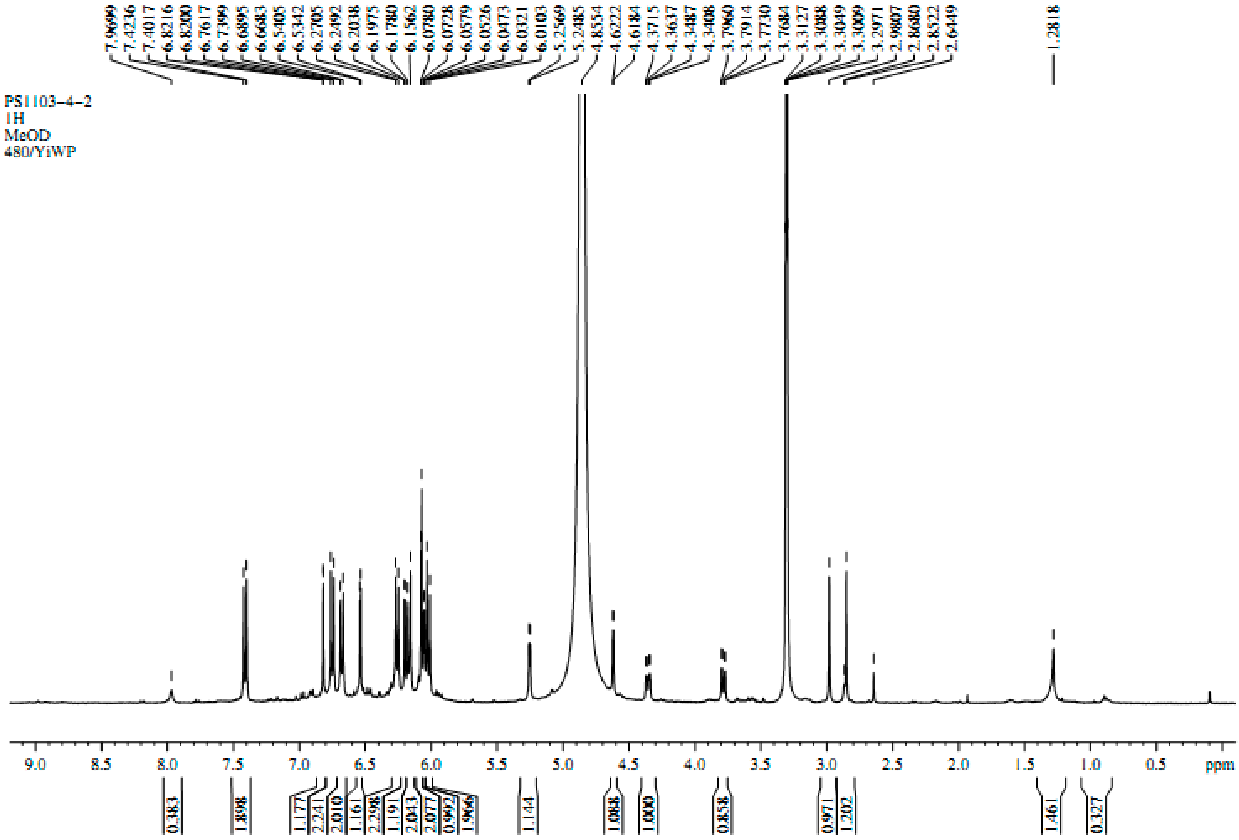

Figure S2. ^{13}C-NMR of **1**.

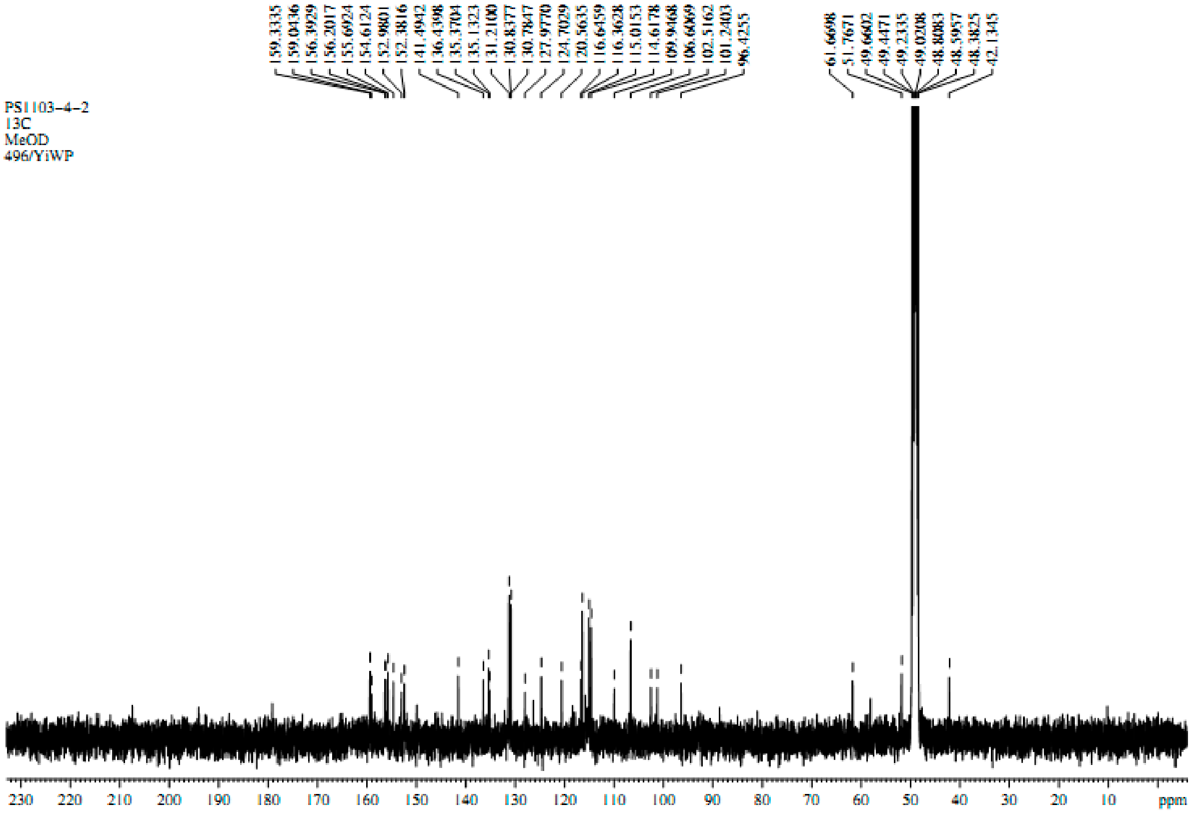

Figure S3. ^{13}C-NMR of **1** (DEPT135).

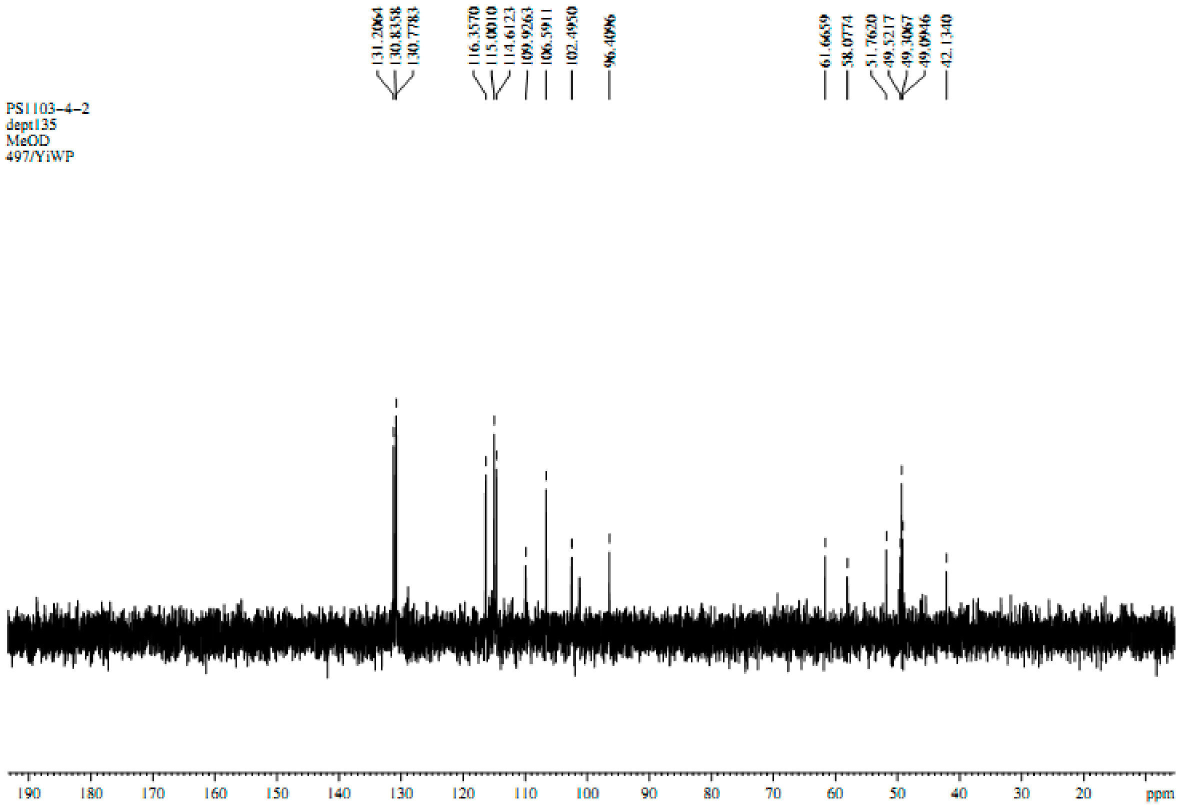

Figure S4. HSQC of **1**.

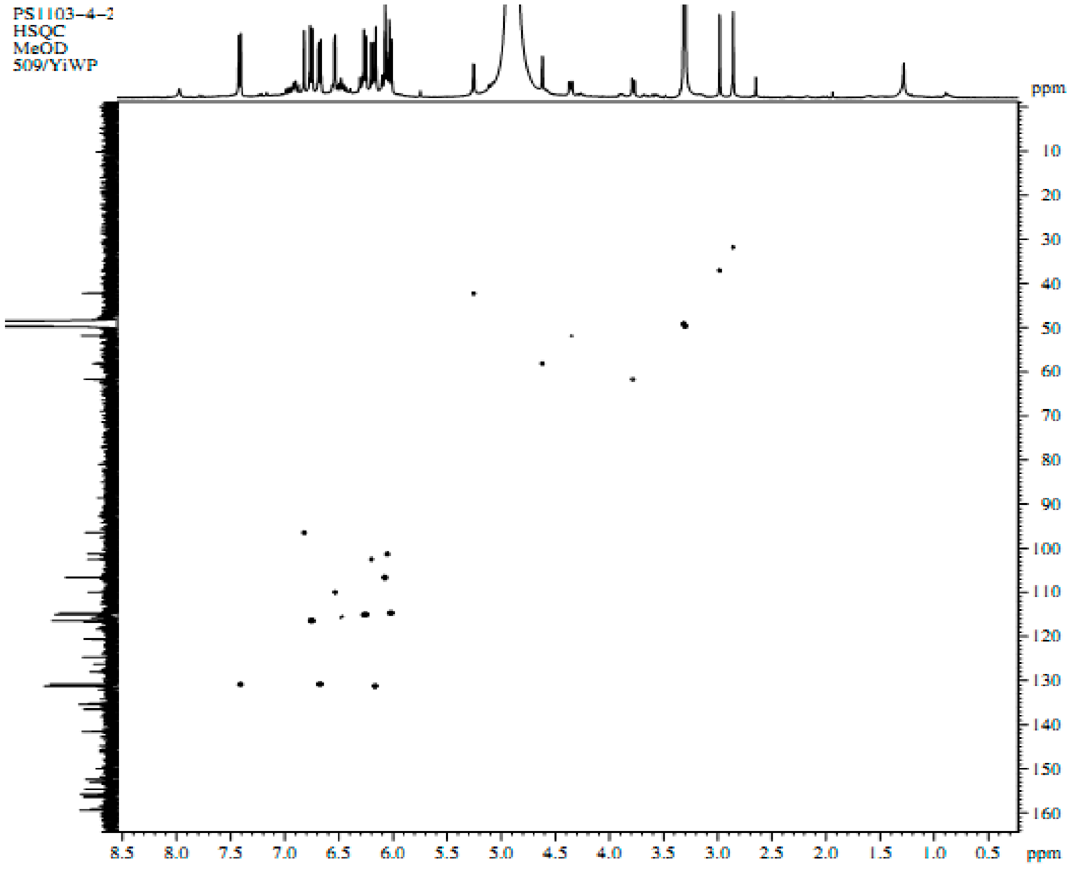

Figure S5. HMBC of **1**.

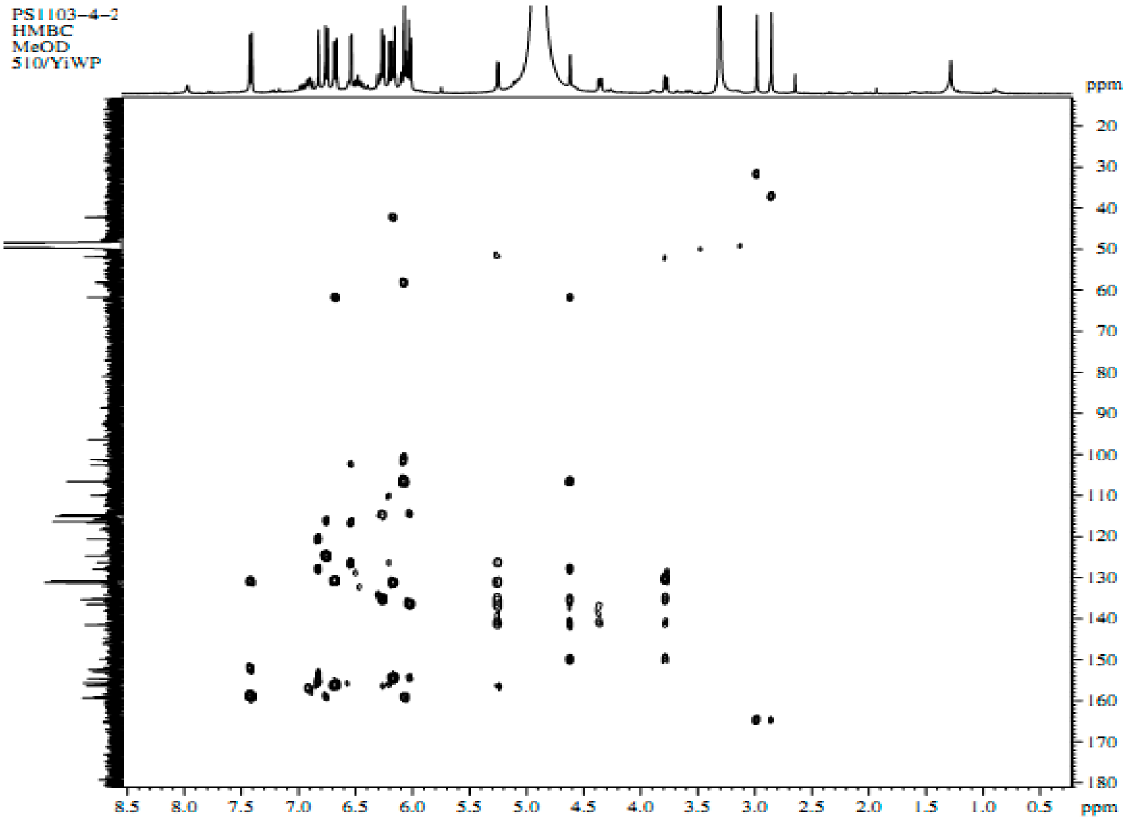

Figure S6. H-H COSYof **1**.

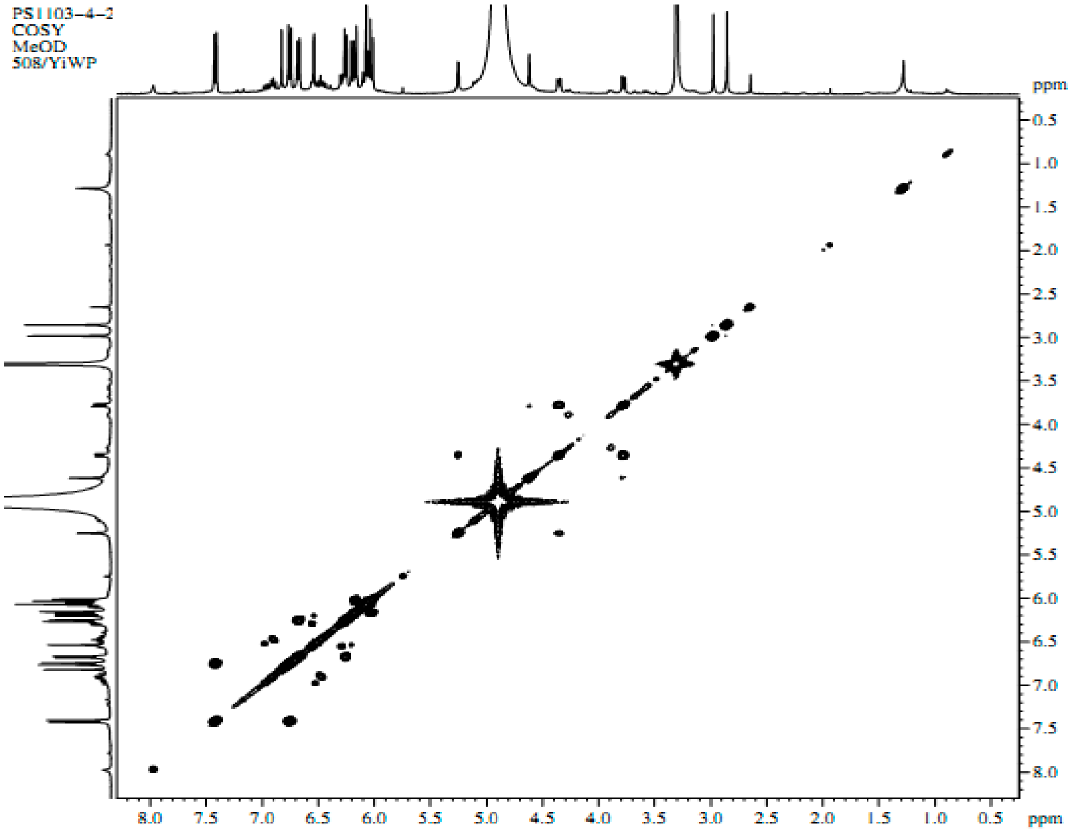

Figure S7. NOESY of **1**.

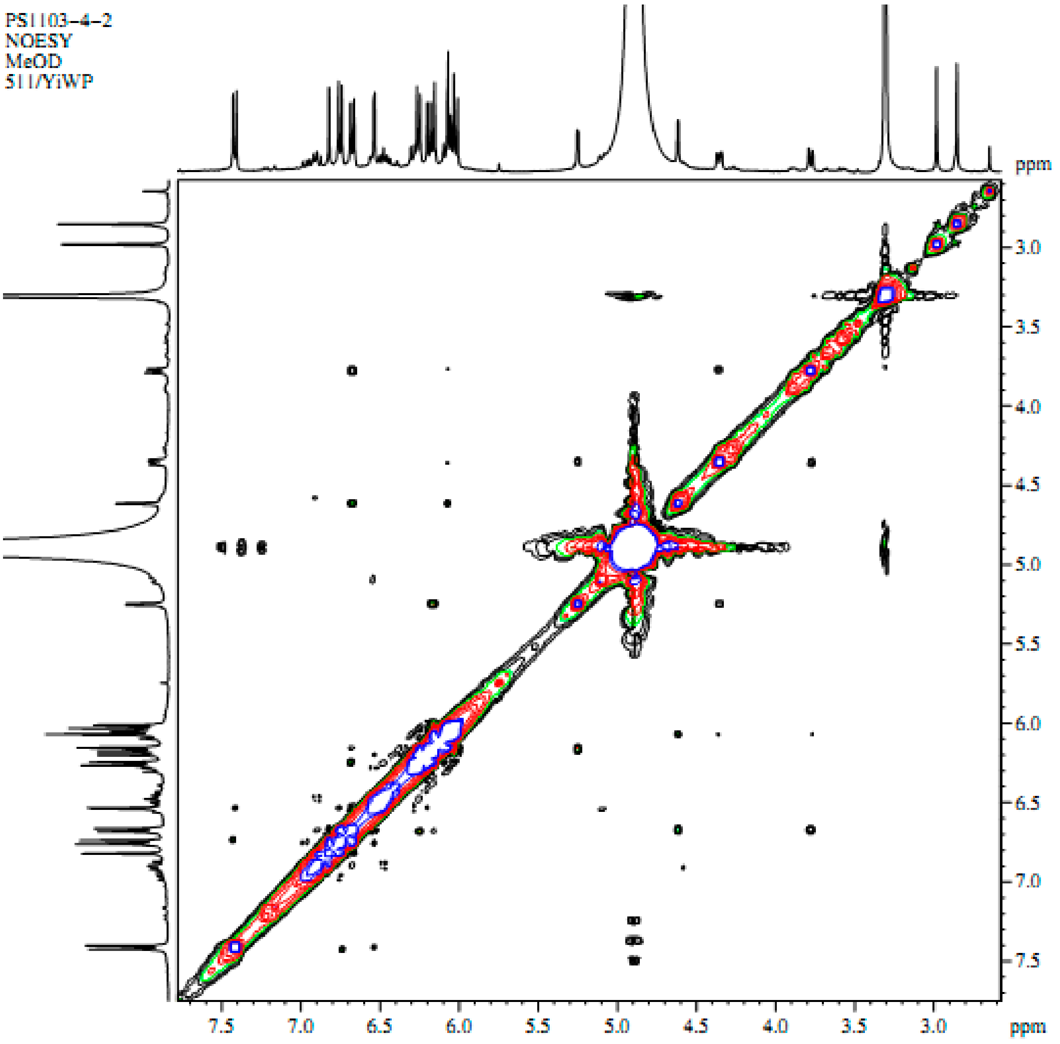

Supplement: Supplementary file 1 [file molecules-19-19549-s001.pdf]
